# Clinical implications of left atrial reverse remodelling after cardiac resynchronization therapy

**DOI:** 10.1093/ehjci/jeac042

**Published:** 2022-02-25

**Authors:** Jan Stassen, Xavier Galloo, Surenjav Chimed, Kensuke Hirasawa, Nina Ajmone Marsan, Victoria Delgado, Pieter van der Bijl, Jeroen J Bax

**Affiliations:** Department of Cardiology, Leiden University Medical Center, Albinusdreef 2, 2300 RC Leiden, The Netherlands; Department of Cardiology, Jessa Hospital, 3500 Hasselt, Belgium; Department of Cardiology, Leiden University Medical Center, Albinusdreef 2, 2300 RC Leiden, The Netherlands; Department of Cardiology, Vrije Universiteit Brussel (VUB), Universitair Ziekenhuis Brussel (UZ Brussel), 1090 Brussels, Belgium; Department of Cardiology, Leiden University Medical Center, Albinusdreef 2, 2300 RC Leiden, The Netherlands; Department of Cardiology, Leiden University Medical Center, Albinusdreef 2, 2300 RC Leiden, The Netherlands; Department of Cardiology, Leiden University Medical Center, Albinusdreef 2, 2300 RC Leiden, The Netherlands; Department of Cardiology, Leiden University Medical Center, Albinusdreef 2, 2300 RC Leiden, The Netherlands; Department of Cardiology, Leiden University Medical Center, Albinusdreef 2, 2300 RC Leiden, The Netherlands; Department of Cardiology, Leiden University Medical Center, Albinusdreef 2, 2300 RC Leiden, The Netherlands; Department of Cardiology, Turku Heart Center, University of Turku and Turku University Hospital, FI-20520 Turku, Finland

**Keywords:** cardiac resynchronization therapy, left atrial reservoir strain, left ventricular global longitudinal strain, mortality

## Abstract

**Aims:**

Left atrial (LA) function is a marker of prognosis in patients with heart failure. The prognostic implications of an improvement in LA function in addition to an improvement in left ventricular (LV) function after cardiac resynchronization therapy (CRT) implantation are unknown. This study aimed to evaluate the prognostic value of a significant change in LA reservoir strain (RS) and/or LV global longitudinal strain (GLS) after initiation of CRT.

**Methods and results:**

LARS and LVGLS were measured with speckle-tracking echocardiography. Significant improvement in LARS and LVGLS was defined as a percentage change of +5% and +20% at 6 months after CRT implantation, respectively. Patients were divided into three groups: no significant reverse remodelling (no improvement in LARS and LVGLS), incomplete reverse remodelling (improvement in LARS or LVGLS), and complete reverse remodelling (improvement in LARS and LVGLS). The primary endpoint was all-cause mortality. A total of 923 patients (mean age 65 ± 10 years, 77% male) were included, of which 221 (24%) had complete reverse remodelling, 414 (45%) incomplete reverse remodelling, and 288 (31%) no significant reverse remodelling. Five-years’ mortality was 24%, 29%, and 36% for patients with complete, incomplete, and no significant reverse remodelling, respectively (*P* < 0.001). On multivariable analysis, complete reverse remodelling (hazard ratio 0.477; 95% confidence interval: 0.362–0.628; *P* < 0.001) was associated with the lowest risk of mortality.

**Conclusions:**

Patients with complete reverse remodelling have a lower mortality risk than those showing incomplete or no significant reverse remodelling. The use of integrated LA and LV deformation imaging may improve risk-stratification of CRT recipients.

## Graphical Abstract

**Graphical Abstract:**
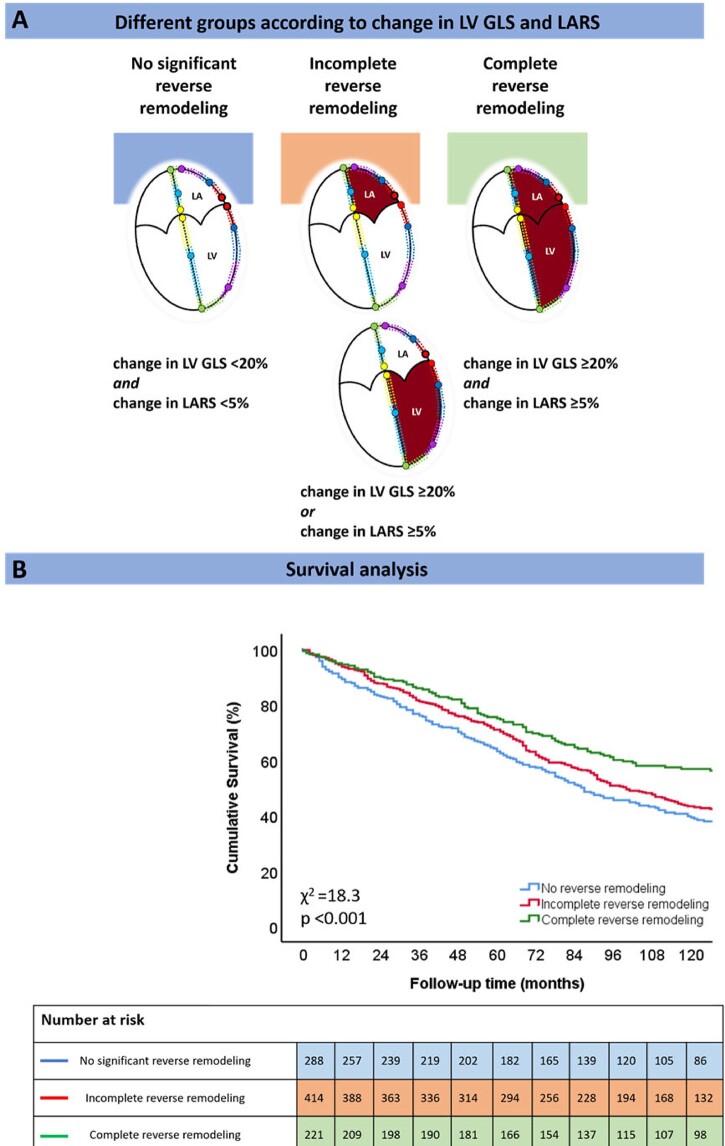
Patients showing complete reverse remodelling (improvement in LARS and LVGLS) have better outcomes than those showing incomplete reverse remodelling (improvement in LARS or LVGLS, but not both) or no significant reverse remodelling (no improvement in LARS and LVGLS).

**See the editorial comment for this article ‘Statistics and individuals’, by Jens-Uwe Voigt, https://doi.org/10.1093/ehjci/jeac067.**

## Introduction

Cardiac resynchronization therapy (CRT) is an established treatment for patients with heart failure (HF) who remain symptomatic despite optimal medical therapy, with a wide QRS complex (≥130 ms) and reduced left ventricular (LV) ejection fraction (EF) (LVEF ≤35%).^1^ In these selected patients, CRT has been shown to alleviate HF symptoms, induce LV reverse remodelling and decrease mortality by resynchronizing the LV.^1^ Previous studies have demonstrated that improvement in LV global longitudinal strain (GLS), which quantifies active myocardial deformation and is a more robust marker of LV performance than LVEF,^2^ is independently associated with improved outcomes in CRT recipients.^3^ In addition, CRT has the potential to reduce functional mitral regurgitation severity^4^ and to improve LV diastolic function,^5,6^ which may lead to left atrial (LA) reverse remodelling.^7,8^ LA reverse remodelling has been linked to better cardiovascular outcomes in patients with HF receiving CRT.^9,10^

Despite evidence that CRT has the ability to improve LA and LV function, the interaction of changes in LA and LV deformation after CRT, as well as their association with outcomes, has not been previously investigated. Accordingly, the aims of this study were (i) to quantify changes in LA reservoir strain (LARS) and LV GLS after CRT and (ii) to assess the prognostic implications of a significant change in LARS, LV GLS or both, 6 months after initiation of CRT.

## Methods

### Patient population and clinical data collection

Patients with HF who underwent CRT implantation according to prevailing guideline recommendations,^1^ were included from an ongoing single-centre registry at the Leiden University Medical Center in The Netherlands. Clinical and echocardiographic data were obtained from the departmental electronic medical record (EPD-vision; Leiden University Medical Center, Leiden, The Netherlands) and retrospectively analysed. An ischaemic aetiology of HF was defined by the presence of significant coronary artery disease on invasive coronary angiography. Quality of life was evaluated with the Minnesota Living with Heart Failure Questionnaire. Renal function was quantified by estimating the glomerular filtration rate with the Modification of Diet in Renal Disease Study (MDRD) equation. The study complies with the Declaration of Helsinki and was approved by the Institutional Review Board. Considering the retrospective nature of the study and all data being handled anonymously, the Medical Ethical Committee waived the need of patient written informed consent.

### Echocardiographic data acquisition and analysis

**Figure 1:**
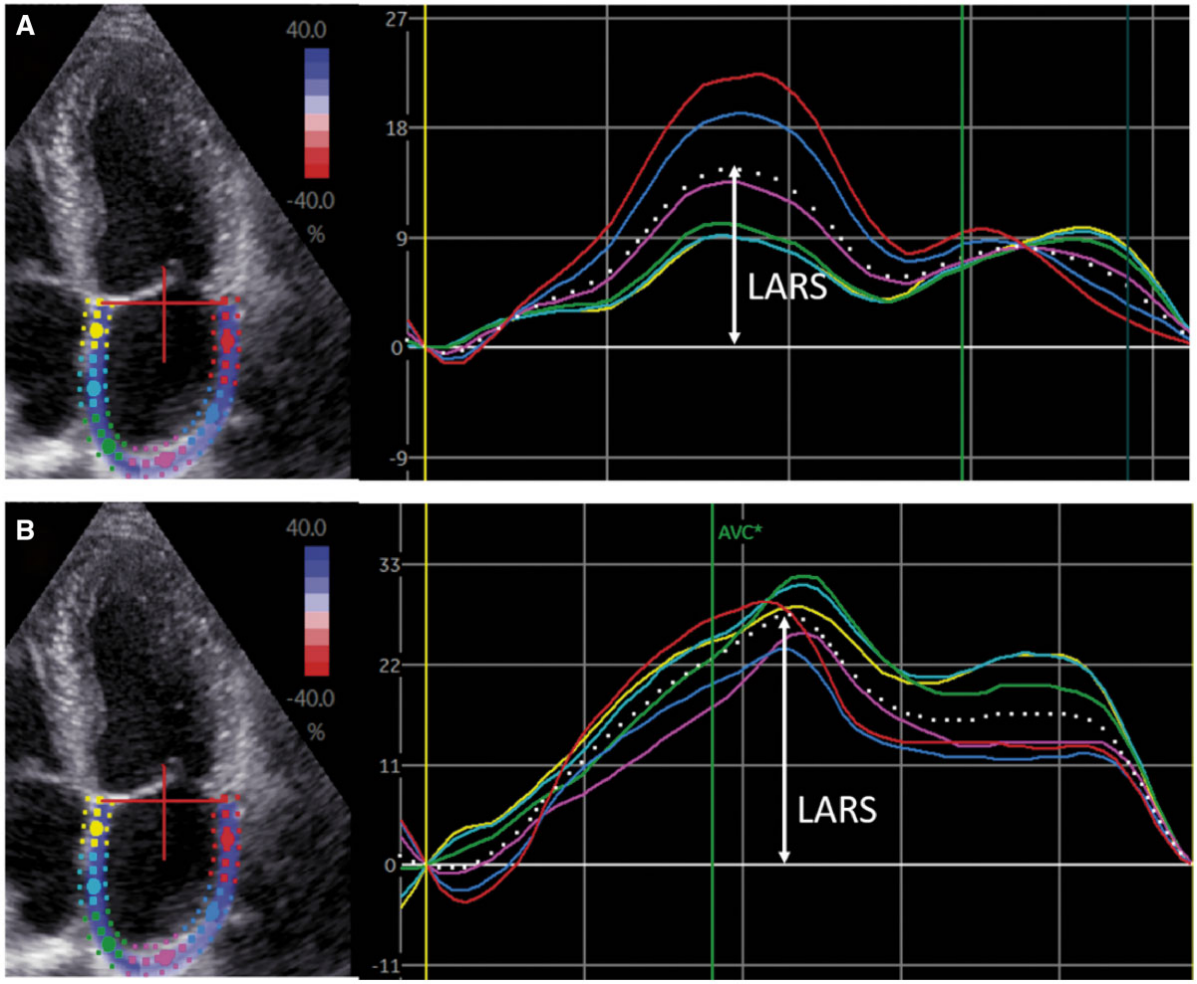
Left atrial speckle tracking strain analysis. LA speckle tracking strain analysis in CRT recipients was performed in an apical four-chamber view. Regional strain vs. time curves are represented by coloured lines, while the average LA strain vs. time curve is represented by the dotted line. LARS is indicated by the arrow. (*A*) Shows the LA strain before CRT implantation (LARS 15%), whereas (*B*) shows the LA strain after CRT implantation (LARS 27%). CRT, cardiac resynchronization therapy; LA, left atrial; LARS, left atrial reservoir strain.

All patients underwent transthoracic echocardiography before CRT implantation in the left lateral decubitus position with commercially available ultrasound equipment (Vivid 7 and E9, GE-Vingmed, Horten, Norway). ECG-triggered echocardiographic data were stored digitally in a cine-loop format for offline analysis using EchoPAC version 203 (GE Medical Systems, Horten, Norway). LV volumes, LVEF and LA volumes were measured using the Simpson’s biplane method.^11^ Right ventricular end-systolic and end-diastolic areas were traced in a focused right ventricular apical view according to current recommendations.^11^ Tricuspid annular plane systolic excursion was measured on M-mode recordings of the lateral tricuspid annulus in a right ventricular-focused view.^11^ Right ventricular peak systolic pressure was derived from the peak velocity of the tricuspid regurgitant jet according to the Bernoulli equation, adding the right atrial pressure (estimated by the inspiratory collapse and diameter of the inferior vena cava).^11^ The severity of mitral and tricuspid regurgitation was graded using a multiparametric approach, as recommended by current guidelines.^12^ Speckle tracking LV GLS was averaged from 17 LV segments, and measured from apical views (two, three, and four chambers).^13^ The region of interest was traced manually and adjusted to the myocardial thickness. Speckle tracking LA strain was measured on the apical four-chamber view, according to current guidelines with the onset of the QRS complex used as the zero-reference point.^14,15^ The endocardium of the LA wall was traced manually and corrected by adjusting the region of interest or the width of the contour, excluding the pulmonary vein ostia and LA appendage (*Figure 1*). LARS was chosen over LA conduit strain and LA contractile strain because it shows a good correlation with LA wall fibrosis on magnetic resonance imaging,^16^ reflects atrial compliance and can still be assessed in patients with atrial fibrillation.^14^ Both LV GLS and LARS are represented as absolute (i.e. positive) values.

### CRT implantation

CRT implantation was performed according to a standard approach, i.e. insertion of the right atrial and ventricular leads via the subclavian or cephalic veins. Before insertion of the LV lead, coronary sinus venography was performed. The LV pacing lead was then introduced into the coronary sinus through an 8 Fr guiding catheter, and positioned in a posterior or posterolateral vein, if possible. Defibrillator functionality was included in most (96%) of the implanted devices. CRT recipients were followed up at regular intervals at the HF outpatient clinic, at which time the device was interrogated. Atrioventricular and interventricular delays were empirically set at 120–140 and 0 ms, respectively. CRT optimization was performed during follow-up visits at the discretion of the treating physician.

### Definition of LV and LA strain response to CRT

**Figure 2:**
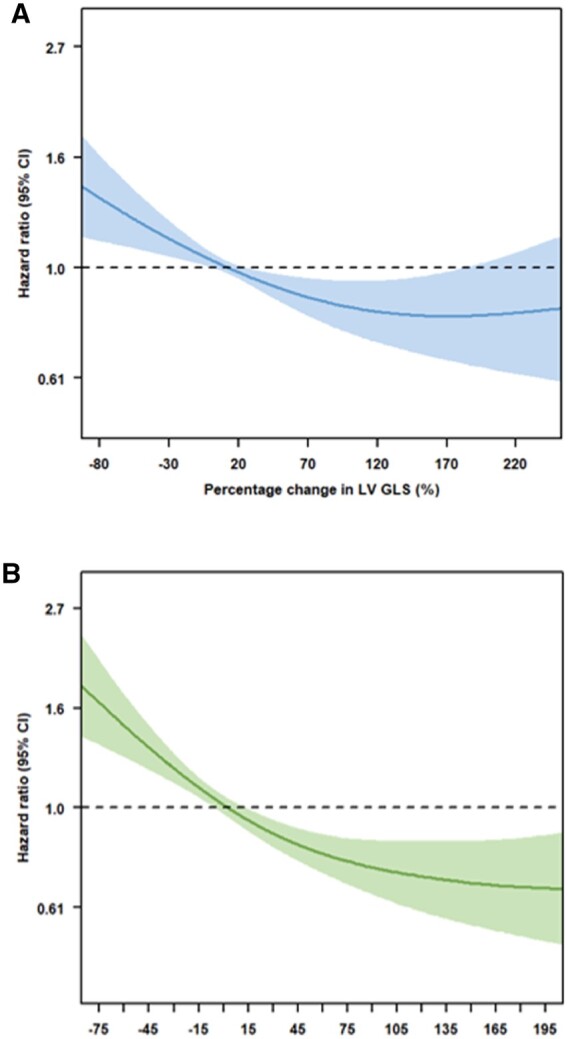
Spline curve for all-cause mortality according to change in LV GLS (*A*) and LARS (*B*). The curves represent the hazard ratio for all-cause mortality with overlaid 95% confidence intervals across the range of percentage change in LV GLS (A) and LARS (B) at 6 months after CRT implantation, compared with pre-implantation values. CRT, cardiac resynchronization therapy; LARS, left atrial reservoir strain; LV GLS, left ventricular global longitudinal strain.

In order to divide the study population into groups according to the degree of LV GLS/LARS change in response to CRT, spline curve analysis was performed. Spline curves were fitted to visualize the relation between the hazard ratio (HR) of all-cause mortality and the echocardiographic parameter in question, i.e. percentage change in LV GLS or LARS 6 months after CRT implantation. Based on this analysis, a 20% change in LV GLS and a 5% change in LARS were identified as optimal cut-off values (i.e. where the predicted HR for all-cause mortality was ≥ 1) (*Figure 2*). The prognostic value of these thresholds was confirmed by Kaplan–Meier survival analysis and differences between groups were analysed using the log-rank test (Supplementary data online, *Figure S1*). The study cohort was subsequently divided into three groups: (i) no significant reverse remodelling (change in LV GLS <20% and change in LARS <5%); (ii) incomplete reverse remodelling (either change in LV GLS ≥20% or change in LARS ≥5%, but not both); and (iii) complete reverse remodelling (change in LV GLS ≥20% and change in LARS ≥5%) (Central Illustration).

### Clinical endpoints

Patients were followed-up for the occurrence of all-cause mortality. Data on mortality were obtained from the departmental cardiology information system (EPD-Vision, Leiden University Medical Center, Leiden, The Netherlands), which is linked to the governmental death registry database. Follow-up data were complete for all patients.

### Statistical analysis

Continuous data are presented as mean ± standard deviation when normally distributed and as median and interquartile range when not normally distributed. Categorical data are presented as frequencies and percentages. Continuous variables were compared using the analysis of variances test with Bonferroni’s *post hoc* analysis when normally distributed, whereas the Kruskal–Wallis test was used to compare continuous variables that did not follow a normal distribution. Categorical variables were compared using the Pearson χ^2^ test. A spline curve analysis was performed to assess the HR for all-cause mortality across a range of LV GLS and LARS values (expressed as percentage change at 6 months after CRT implantation, compared with pre-implantation values). A change in LV GLS of +20% and a change in LARS of +5% were identified, based on mortality excess (i.e. in which the predicted HR was ≥1). The inter- and intra-observer variabilities of LARS measurement were assessed by calculating the intra-class correlation coefficient on 20 randomly selected patients. The intra-class correlation coefficients for inter- and intra-observer variability were 0.94 [95% confidence interval (CI): 0.85–0.97, *P* < 0.001] and 0.95 (95% CI: 0.87–0.98, *P* < 0.001), respectively. The intra-class correlation coefficients for inter- and intra-observer variability of LV GLS have been published previously,^17^ with an intra-class correlation coefficient for inter- and intra-observer variability of 0.92 (95% CI: 0.84–0.97, *P* < 0.001) and 0.97 (95% CI: 0.89–0.99, *P* < 0.001), respectively, showing excellent agreement. Event-free survival curves were generated using the Kaplan–Meier method and differences between the three groups (no significant reverse remodelling, incomplete reverse remodelling and complete reverse remodelling) were analysed using the log-rank test. To assess the association of different groups of LV GLS and LARS change with all-cause mortality, uni- and multivariable Cox proportional hazard models were constructed. For both uni- and multivariable analyses, HRs with 95% CIs were calculated. To inspect for multicollinearity, the Pearson correlation coefficient was calculated between continuous variables, assuming no significant multicollinearity when the correlation coefficient was <50%. In addition, the variation inflation factor was also calculated, assuming no significant multicollinearity when this value was <5. To investigate the incremental value of significant change(s) in LV GLS and/or LARS over clinical and conventional echocardiographic parameters to predict outcome, likelihood ratio testing was performed, for which the change in global chi-square values was calculated and reported. A two-sided *P*-value <0.05 was considered statistically significant. Statistical analysis was performed using SPSS for Windows, version 25.0 (IBM, Armonk, NY, USA) and R version 4.0.1 (R Foundation for Statistical Computing, Vienna, Austria).

## Results

### Clinical and echocardiographic characteristics at baseline

A total of 923 patients (mean age 65 ± 10 years, 77% male) were included. Baseline clinical characteristics are shown in *Table 1*, while *Table 2* summarizes the echocardiographic data for the overall population. An ischaemic aetiology of HF was present in 539 (58.4%) patients. The mean LV GLS and median LARS at baseline were 7.6 ± 3.4% and 12.7 (7.8–18.4)%, respectively.

**Table 1:** Baseline clinical characteristics

	Overall population	No significant reverse remodelling	Incomplete reverse remodelling	Complete reverse remodelling	*P*-value
(*n* = 923)	(*n* = 288)	(*n* = 414)	(*n* = 221)		
Age (years)	65.4 (±10.4)	65.5 (±10.3)	65.4 (±10.4)	65.2 (±10.6)	0.962
Male sex (%)	706 (76.5%)	224 (77.8%)	329 (79.5%)	153 (69.2%)^†^	0.012
Arterial hypertension (%)	438 (47.5%)	132 (45.8%)	193 (46.6%)	113 (51.1%)	0.428
Diabetes mellitus (%)	195 (21.1%)	68 (23.6%)	88 (21.3%)	39 (17.6%)	0.262
Dyslipidaemia (%)	397 (43.0%)	128 (44.4%)	185 (44.7%)	84 (38.0%)	0.214
Current smoker (%)	148 (16.0%)	49 (17.0%)	73 (17.6%)	26 (11.8%)	0.162
BMI (kg/m^2^)	26.6 (±4.2)	27.0 (±4.3)	26.4 (±4.2)	26.2 (±4.1)	0.121
Ischaemic aetiology (%)	539 (58.4%)	194 (67.4%)	247 (59.7%)	98 (44.3%)^*^^†^	<0.001
QoL score	29.5 (16.0–45.0)	33.0 (17.0–48.5)	26.0 (15.0–42.0)^*^	32.0 (18.0–44.5)	0.030
6MWT, m	337.4 (±118.4)	327.7 (±116.4)	339.8 (±122.2)	346.0 (±113.5)	0.271
NYHA III–IV (%)	592 (64.1%)	187 (64.9%)	258 (62.3%)	147 (66.5%)	0.394
Sinus rhythm	682 (73.9%)	146 (66.1%)	303 (73.2%)	233 (80.9%)^*^	0.004
QRS duration (ms)	154 (±35)	148 (±34)	153 (±34)	164 (±34)^*^^†^	<0.001
Beta-blocker (%)	684 (74.1%)	214 (74.3%)	310 (74.9%)	160 (72.4%)	0.790
ACE-i/ARB (%)	821 (88.9%)	263 (91.3%)	366 (88.4%)	192 (86.9%)	0.255
MRA (%)	386 (41.8%)	118 (41.0%)	185 (44.7%)	83 (37.6%)	0.209
Diuretics (%)	724 (78.4%)	235 (81.6%)	329 (79.5%)	160 (72.4%)^*^	0.035
Statin (%)	575 (62.3%)	185 (64.2%)	270 (65.2%)	120 (54.3%)^†^	0.018
eGFR (mL/min/1.73 m^2^)	67.4 (±23.7)	66.2 (±25.9)	67.2 (±22.6)	69.6 (±22.6)	0.271
Haemoglobin (g/dL)	13.4 (±1.6)	13.2 (±1.6)	13.5 (±1.6)	13.5(±1.6)^*^	0.023

Values are presented as mean ± SD, median (IQR), or *n* (%).

ACE-i, angiotensin-converting enzyme inhibitor; ARB, angiotensin receptor blocker; BMI, body mass index; eGFR, estimated glomerular filtration rate; MRA, mineralocorticoid receptor antagonist; MWT, minute walking test; NYHA, New York Heart Association; QoL, quality of life.

* *P* < 0.05 vs. no significant reverse remodelling.

† *P* < 0.05 vs. incomplete reverse remodelling.

**Table 2:** Baseline echocardiographic characteristics

	Overall population	No significant reverse remodelling	Incomplete reverse remodelling	Complete reverse remodelling	*P*-value
(*n* = 923)	(*n* = 288)	(*n* = 414)	(*n* = 221)		
LV EDV (mL)	189 (149–243)	181 (145–237)	189 (151–244)	199 (146–250)	0.223
LV ESV (mL)	138 (102–179)	129 (97–172)	138 (104–176)	145 (106–190)^*^	0.027
LVEF (%)	27.7 (±8.0)	29.1 (±7.5)	27.5 (±8.2)^*^	26.2 (±7.9)^*^	<0.001
LV GLS (%)	7.6 (±3.4)	8.6 (±3.4)	7.7 (±3.3)^*^	6.0 (±2.8)^*^^†^	<0.001
LAVi (mL/m^2^)	43 (±19)	43 (±22)	43 (±17)	43 (±18)	0.921
LARS (%)	12.7 (7.8–18.4)	15.9 (10.0–21.4)	12.2 (7.5–17.8)^*^	10.2 (6.3–15.3)^*^^†^	<0.001
Moderate or severe MR (%)	340 (36.8%)	101 (35.1%)	150 (36.2%)	89 (40.3%)	0.505
RVEDA (cm^2^)	22.3 (±6.9)	22.4 (±7.2)	22.1 (±6.8)	22.4 (±7.0)	0.817
RVESA (cm^2^)	14.3 (±6.1)	14.3 (±6.2)	14.2 (±5.9)	14.5 (±6.3)	0.802
TAPSE (mm)	16.4 (±4.7)	16.7 (±4.8)	16.3 (±4.4)	16.3 (±5.2)	0.481
RA area (cm^2^)	17.7 (14.2–22.7)	17.7 (14.3–22.4)	17.7 (14.4–22.0)	17.6 (14.0–23.8)	0.916
TR velocity (m/s)	2.6 (±0.6)	2.6 (±0.6)	2.6 (±0.6)	2.6 (±0.6)	0.457
PASP	35.0 (±13.8)	35.2 (±15.0)	34.4 (±12.8)	35.9 (±13.9)	0.530
Moderate or severe TR (%)	177 (19.2%)	51 (17.7%)	75 (18.1%)	51(23.1%)	0.116

Values are presented as mean ± SD, median (IQR), or *n* (%).

EDA, end-diastolic area; EDV, end-diastolic volume; EF, ejection fraction; ESA, end-systolic area; ESV, end-systolic volume; GLS, global longitudinal strain; LARS, left atrium reservoir strain; LAVi, left atrial volume index; LV, left ventricle; PASP, pulmonary artery systolic pressure; RA, right atrium; TR, tricuspid regurgitation; RV, right ventricle; TAPSE, tricuspid annular plane systolic excursion.

* *P* < 0.05 vs. no significant reverse remodelling.

† *P* < 0.05 vs. incomplete reverse remodelling.

### Changes in LV GLS and LARS after CRT

At 6 months after CRT implantation, 288 (31.2%) patients showed no significant reverse remodelling, while 414 (44.9%) showed incomplete reverse remodelling and 221 (23.9%) showed complete reverse remodelling. Of the 414 individuals with incomplete reverse remodelling, a significant improvement in LV GLS without a significant change in LARS was seen in 270 (65.2%) patients, while 144 (34.8%) patients showed a significant change in LARS but not in LV GLS. The Pearson correlation coefficient for the relative change in LARS and LV GLS (expressed as continuous variables) was 0.153, whereas the variation inflation factor was 1, assuming no significant multicollinearity between both variables.

Patients with complete reverse remodelling were more likely to be female with a non-ischaemic HF aetiology, more likely to be in sinus rhythm and had a longer QRS duration at baseline, compared with the other two groups (*Table 1*). In addition, patients with complete reverse remodelling had larger LV ESV, lower LVEF and more impaired LV GLS and LARS at baseline, compared with the other two groups (*Table 2*).

Of the 340 patients with moderate or severe MR at baseline, 200 (59%) patients showed significant improvement in MR severity (reduction of ≥1 grade) at follow-up, whereas 140 (41%) patients showed no significant improvement (reduction of <1 grade). The percentage change in LV GLS for MR improvers vs. non-improvers was 43 ± 10% vs. 24 ± 8%, respectively (*P* = 0.178), whereas the percentage change in LARS was 46 ± 8% vs. 10 ± 5%, respectively (*P* < 0.001).

### LV GLS and LARS improvement after CRT: prognostic implications

**Figure 3:**
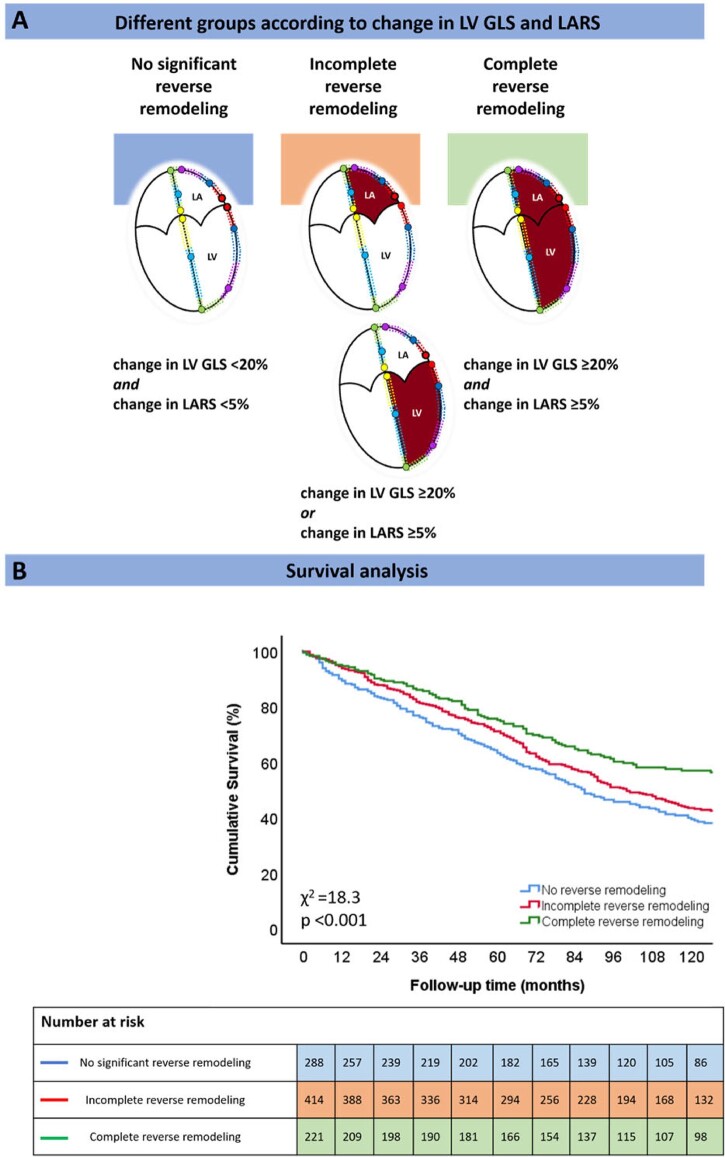
Kaplan–Meier curve for time to cumulative survival, according to the three different study groups. LA, left atrium; LARS, left atrial reservoir strain; LV, left ventricle; LV GLS, left ventricular global longitudinal strain.

After a median follow-up of 91 (49–138) months, 546 (59.2%) patients died. Five-year mortality rates were 36%, 29%, and 24% for patients with no significant reverse remodelling, incomplete reverse remodelling, and complete reverse remodelling, respectively (*P* < 0.001) (*Figure 3*). Patients with complete reverse remodelling showed significantly lower mortality rates compared with those with incomplete reverse remodelling and no significant reverse remodelling (*P* = 0.002 and *P* < 0.001, respectively). On multivariable analysis, incomplete reverse remodelling (HR: 0.717; 95% CI: 0.585–0.878; *P* = 0.001) and complete reverse remodelling (HR: 0.477; 95% CI: 0.362–0.628; *P* < 0.001) were independently associated with better outcomes (*Table 3*). There was no interaction between the percentage difference in LV GLS and change in LV ESV with outcome (*P* value = 0.709) or the percentage difference in LARS and change in LV ESV with outcome (*P* value = 0.092).

**Table 3:** Uni- and multivariable Cox regression analyses

	Univariable analysis		Multivariable analysis	
HR (95% CI)	*P*-value	HR (95% CI)	*P*-value
Age	1.050 (1.040–1.060)	<0.001	1.033 (1.022–1.044)	<0.001
Male sex	1.506 (1.217–1.863)	<0.001	1.306 (1.039–1.643)	0.022
Arterial hypertension	1.092 (0.922–1.293)	0.306		
Diabetes mellitus	1.499 (1.233–1.822)	<0.001	1.229 (0.999–1.513)	0.051
Dyslipidaemia	1.226 (1.036–1.452)	0.018	1.021 (0.851–1.224)	0.825
Ischaemic aetiology for heart failure	1.598 (1.339–1.907)	<0.001	1.181 (0.965–1.445)	0.107
eGFR (mL/min/1.73 m^2^)	0.977 (0.974–0.981)	<0.001	0.988 (0.984–0.992)	<0.001
NYHA III–IV	1.785 (1.473–2.162)	<0.001	1.310 (1.071–1.603)	0.009
Atrial fibrillation	1.108 (0.980–1.253)	0.101		
LAVi baseline (mL/m^2^)	1.015 (1.012–1.019)	<0.001	1.006 (1.001–1.010)	0.010
LARS baseline (%)	0.941 (0.929–0.953)	<0.001	0.958 (0.943–0.974)	<0.001
LV GLS baseline (%)	1.048 (1.021–1.076)	<0.001	0.976 (0.949–1.004)	0.097
TAPSE (mm)	0.948 (0.930–0.966)	<0.001	0.979 (0.960–0.998)	0.031
LV ESV reduction ≥15%	0.680 (0.574–0.804)	<0.001	0.824 (0.674–1.008)	0.060
Absolute increase in LVEF ≥5%	0.705 (0.596–0.834)	<0.001	0.853 (0.698–1.044)	0.123
No significant reverse remodelling	Reference group		Reference group	
Incomplete reverse remodelling	0.849 (0.703–1.025)	0.089	0.717 (0.585–0.878)	0.001
Complete reverse remodelling	0.598 (0.471–0.759)	<0.001	0.477 (0.362–0.628)	<0.001

eGFR, estimated glomerular filtration rate; ESV, end-systolic volume; GLS, global longitudinal strain; LARS, left atrial reservoir strain; LAVi, left atrium volume index; LV, left ventricular; NYHA, New York Heart Association; TAPSE, tricuspid annular plane systolic excursion.

Of interest, when looking at the percentage change in LARS and LV GLS expressed as continuous variables, a percentage change in LARS (HR: 0.997; 95% CI: 0.995–0.998; *P* < 0.001) as well as a percentage change in LV GLS (HR: 0.998; 95% CI 0.996–0.999; *P* = 0.004) were both independently associated with all-cause mortality on the multivariable Cox regression analysis.

### Incremental prognostic value of improved LV GLS and LARS

In order to investigate the incremental prognostic value of change in LV GLS and LARS, in addition to the conventional definition of CRT response (i.e., ≥15% reduction in LV ESV at 6 months’ follow-up after CRT implantation^18^) and various clinical parameters, likelihood ratio testing was performed. The baseline model comprised all covariates used in the multivariable Cox regression analysis, i.e. age, sex, arterial hypertension, diabetes mellitus, dyslipidaemia, ischaemic aetiology for HF, estimated glomerular filtration rate, New York Heart Association class III–IV, baseline LV ESV, baseline left atrium volume index, baseline LV GLS, baseline LARS, and tricuspid annular plane systolic excursion.

**Figure 4:**
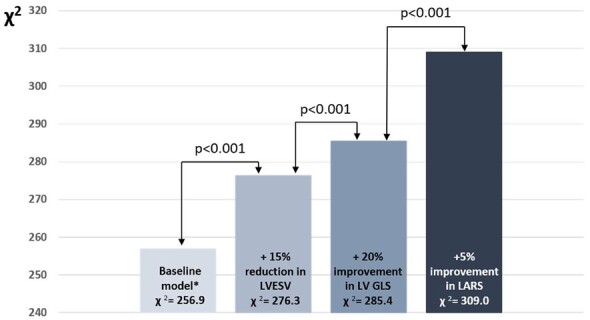
Likelihood ratio test for the incremental prognostic value of a significant change in LV GLS and LARS. The addition of a 20% change in LV GLS and a 5% change in LARS to a baseline clinical model is associated with significant increases in the χ^2^ value. *The baseline model includes age, sex, arterial hypertension, diabetes mellitus, dyslipidaemia, ischaemic aetiology for heart failure, estimated glomerular filtration rate, New York Heart Association functional class III–IV, baseline left ventricular end-systolic volume, baseline left atrium volume index, baseline left atrial reservoir strain, baseline left ventricular global longitudinal strain, and tricuspid annular plane systolic excursion. LARS, left atrial reservoir strain; LV ESV, left ventricular end-systolic volume; LV GLS, left ventricular global longitudinal strain.

Addition of the conventional definition of CRT response (≥15% reduction in LV ESV) to the baseline clinical model showed incremental prognostic value (*P* < 0.001) (*Figure 4*).

Next, addition of a significant change in LV GLS (change in LV GLS ≥20%) to the baseline clinical model and ≥15% reduction in LV ESV, yielded incremental prognostic value (*P* < 0.001) (*Figure 4*). A third model, including both a significant improvement in LV GLS and LARS (change in LV GLS ≥20% and change in LARS ≥5%), provided further incremental prognostic value (*P* < 0.001) (*Figure 4*).

## Discussion

The main findings of this study are summarized as follows: (i) patients with complete reverse remodelling (significant improvement in both LARS and LVGLS) after CRT implantation have better outcomes than patients showing incomplete reverse remodelling (significant improvement in LARS or LVGLS) or no significant reverse remodelling (no significant improvement in LARS and LVGLS) and (ii) an integrated assessment of changes in LARS and LV GLS has incremental prognostic value over conventional echocardiographic indices of LV reverse remodelling.

### Change in LV GLS after CRT

The echocardiographic definition of CRT response is most commonly defined as ≥15% reduction in LV ESV at 6 months after device implantation.^19^ The use of LV ESV to define CRT response however, has some limitations: (i) it does not adequately reflect active myocardial deformation and (ii) a reduction in LV ESV may occur without the recruitment of contractile reserve, which has been associated with better outcomes in patients receiving CRT.^20^ Myocardial strain imaging by speckle tracking echocardiography at least partially overcomes these limitations, and its use in characterizing CRT response is supported by outcome data. In a study of 761 HF patients, Pouleur *et al.*^3^ demonstrated that CRT resulted in a significant improvement in LV function assessed by LV GLS and this improvement translated into better outcomes. In addition, van der Bijl *et al.*^17^ demonstrated that patients showing a significant improvement in LV GLS but not in LV ESV had better long-term outcomes when compared with patients showing no improvement in LV GLS and LV ESV. The results of this study further support the use of LV GLS in patients receiving CRT and show the incremental value of LV GLS to predict outcomes over conventional echocardiographic parameters of CRT response.

### Change in LARS after CRT

LV diastolic dysfunction is a common finding in HF with reduced LVEF and may lead to elevated LV filling pressures, which increase LA afterload. In addition, functional mitral regurgitation is frequently found in HF patients,^4^ and may impose an additional volume overload on the thin-walled LA, leading to LA structural remodelling.^21^ LA remodelling is accompanied by an increase in interstitial fibrosis of the atrial wall, leading to a progressive reduction in LA compliance. Reduced LA compliance in turn, not only decreases LV preload (and therefore LV cardiac output)^22^ but also unfavourably increases the pulsatile load on the pulmonary circulation, contributing to the development of post-capillary pulmonary hypertension and right ventricular-pulmonary arterial uncoupling.^23,24^ LA electrical and structural remodelling also enhances the risk of developing atrial fibrillation^22^ which has been associated with poor outcomes in HF.^25^ CRT has the potential to reverse many of these deleterious effects on the LA. The initiation of CRT significantly reduced LA volume in a study of 107 patients with HF.^7^ Moreover, LA reverse remodelling after CRT has been associated with a significant reduction in the risk of incident atrial arrhythmias,^26^ as well as a reduction in the occurrence of HF hospitalization or death.^27^

Although the beneficial effect of CRT on LA volume is well described, LA functional changes occur well before LA dilatation occurs.^28^ Furthermore, LARS is a functional parameter and has shown a good correlation with LA compliance. LARS has also shown a good correlation with the extent of atrial fibrosis on cardiac magnetic resonance imaging.^16^ The prognostic impact of a significant change in LARS after CRT implantation, however, has not been extensively evaluated in CRT recipients. In a study of 30 patients undergoing CRT implantation, Valzania *et al.*^8^ showed an improvement of LARS in CRT responders, with concomitant improvement in LV systolic and diastolic function. In a limited cohort of CRT recipients, Dokuni *et al.*^9^ investigated the effects of CRT on LARS and demonstrated an association between improved LARS after CRT and better outcomes.

### Change in LV GLS and LARS: implications for CRT recipients

Because the LA is anchored by the pulmonary veins, the major determinant of LA expansion is the systolic descent of the atrioventricular plane towards the apex, which is mainly driven by LV longitudinal function. Impaired LV longitudinal function and loss of LV synchrony, which is typically seen in CRT candidates, may therefore have a negative impact on LA synchrony and LA reservoir function. Dokuni *et al.*^9^ indeed demonstrated that CRT recipients show impaired LA reservoir function in parallel with LA dyssynchrony, becoming more pronounced as the QRS complex broadens. This shows that LA and LV (dys)function are closely linked in CRT candidates. In patients with impaired LA reservoir function, CRT may therefore have the potential to improve LA function by reversing LV dyssynchrony and subsequently, LA dyssynchrony. The results of the present study underscore the importance of evaluating both LA and LV function by showing that complete left-sided reverse remodelling is associated with better outcomes compared with incomplete reverse remodelling or no significant reverse remodelling.

Interestingly, patients who showed complete reverse remodelling initially had lower LA and LV strain values. This might at least partly be explained by the recruitment of contractile reserve. The presence of contractile reserve in CRT candidates has been demonstrated with dobutamine stress-echocardiography and was associated with better outcomes.^20,29^ As such, patients with contractile reserve, may initially have lower LA and LV strain values, but show the ability to have a more pronounced improvement in LA and LV strain values after CRT implantation. Additional studies however, are needed to confirm this hypothesis.

### Clinical implications of LARS and LV GLS change after CRT

The assessment of LARS and LV GLS after CRT implantation may allow the identification of a subgroup of CRT recipients who might otherwise be classified as responders by conventional criteria, but who will nevertheless experience suboptimal outcomes. The identification of such patients may argue for more intensive follow-up and allow the institution of therapies to optimize the effects of CRT, especially on LA and LV mechanics.^30^ For example, more recently introduced HF treatments, such as sodium-glucose co-transport 2 inhibitors and angiotensin-neprilysin inhibitors have shown promising effects on LV longitudinal function.^31,32^ In patients with atrial fibrillation, maintenance of sinus rhythm by pharmacological therapy or catheter ablation may improve LA reverse remodelling.^33^ In patients with significant residual mitral regurgitation after CRT implantation, percutaneous mitral valve repair represents another potential approach to improve LA function.^34^ However, whether these strategies will translate into better clinical outcomes in CRT recipients, requires prospective, randomized trials.

In addition, although we often use a reduction in LV ESV ≥15% to define an echocardiographic response to CRT, this study shows that assessing the change in LARS and LV GLS shows incremental prognostic value over this parameter. Whether LARS and LV GLS should replace the well-established parameter of a ≥15% reduction in LV ESV however, requires further research.

### Study limitations

This study is subject to the limitations of its single-centre, retrospective, and observational design. Patients who died during the first 6 months after CRT implantation could not be included, and may have caused survival bias. LARS and LV GLS are vendor-dependent parameters, which cannot be compared directly between different platforms, and the thresholds of LARS and LV GLS used to define a CRT response in this study may not be generalizable to all patient populations. The duration of echocardiographic follow-up (6 months) disallows conclusions on whether LARS will potentially improve even further in some CRT recipients. Data on HF hospitalizations were not available. Mortality was ascertained by review of hospital records, linked to the governmental death registry database, which does not include granular detail on cardiac vs. non-cardiac mortality.

## Conclusion

Improvement in LV GLS and/or LARS at 6 months post-CRT is independently associated with a lower risk of mortality. Patients with complete reverse remodelling have the lowest mortality risk when compared with patients showing incomplete or no significant reverse remodelling. This supports the use of integrated LA and LV deformation imaging to assess CRT response and may aid in the refinement of risk-stratification of patients treated with CRT.

## Supplementary data

Supplementary data are available at *European Heart Journal - Cardiovascular Imaging* online.

## Funding

J.S. received funding from the European Society of Cardiology (ESC Training Grant App000064741).

**Conflict of interest:** The Department of Cardiology, Heart Lung Center, Leiden University Medical Centre received research grants from Abbott Vascular, Bayer, Biotronik, Bioventrix, Boston Scientific, Edwards Lifesciences, GE Healthcare, Ionis, and Medtronic. J.J.B. received speaker fees from Abbott Vascular. N.A.M. received speaker fees from Abbott Vascular and GE Healthcare. V.D. received speaker fees from Abbott Vascular, Edwards Lifesciences, GE Healthcare, Medtronic, MSD, and Novartis. The remaining authors have nothing to disclose.

## Data availability

The data underlying this article will be shared on reasonable request to the corresponding author.

## Supplementary Material

jeac042_Supplementary_Data
