# Supplementary material for: Clinical implications of left atrial reverse remodelling after cardiac resynchronization therapy
Source: Eur Heart J Cardiovasc Imaging. 2022 Feb 25;23(6):730–40. doi: 10.1093/ehjci/jeac042 (PMC9291382; doi:10.1093/ehjci/jeac042)
Supplement: jeac042_Supplementary_Data [file jeac042_supplementary_data.docx]

**
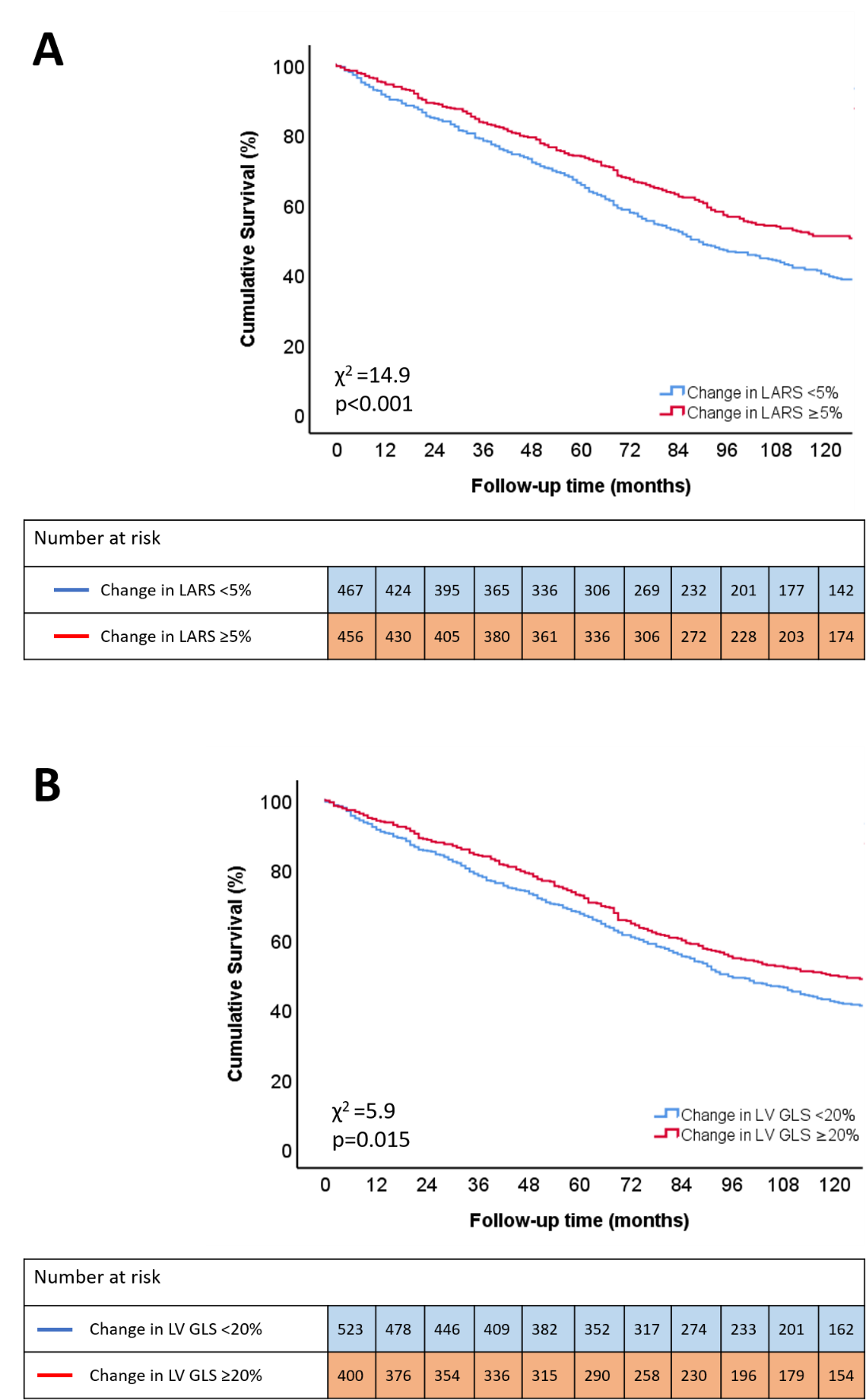
Figure S1 – Kaplan-Meier curves for time to cumulative survival, according to percentage change in LARS (A) and percentage change in LV GLS (B).**

LARS = left atrial reservoir strain; LV GLS = left ventricular global longitudinal strain.
